# Enhancing ovarian cancer care: a systematic review of guideline adherence and clinical variation

**DOI:** 10.1186/s12889-019-6633-4

**Published:** 2019-03-12

**Authors:** Kahren M. White, Holly Seale, Reema Harrison

**Affiliations:** 1 School of Public Health and Community Medicine, University of New South Wales, Sydney, Australia; 2 Cancer Institute NSW, PO Box 41, Alexandria, NSW 1435 Australia

**Keywords:** Unwarranted clinical variation, Ovarian cancer, Quality improvement, Effective care

## Abstract

**Background:**

Clinical variation in ovarian cancer care has been reported internationally. Using Wennberg’s classification of clinical variation as effective care we can conceptualise variation through deviation from clinical guidelines. The aim of this review was to address knowledge gaps in the effectiveness of attempts to reduce unwarranted clinical variation through addressing the following questions: What is the evidence of guideline adherence in ovarian cancer and its deviation?; what are the key factors associated with variation in guideline adherence in ovarian cancer care?; and what quality improvement approaches have been used and what is the evidence of their effectiveness in enhancing guideline adherence in ovarian cancer care?.

**Methods:**

Keywords and synonyms for the major concepts of ovarian cancer, guideline adherence and safety were developed and combined to form the search strategy. Systematic searches of four electronic databases were undertaken of publications from January 2007 to November 2018. Retrieved articles were assessed against the eligibility criteria to determine those for inclusion.

**Results:**

Thirty-two papers were included in the review with three broad groupings identified: adherence to and deviation from guidelines (either local, national or international guidelines); factors impacting guidelines adherence; and quality improvement approaches.

**Conclusions:**

Unwarranted clinical variation may be used as a marker for the effectiveness of a health system, based on the outcome of this systematic review. This review found that the implementation of quality indicators through a formal quality improvement program lead to improvements in guideline adherent care. Further research on outcomes of implementing quality improvement programs in ovarian cancer care will improve the ability to implement centralised care and further identify factors that to improve outcomes in ovarian cancer care.

**Electronic supplementary material:**

The online version of this article (10.1186/s12889-019-6633-4) contains supplementary material, which is available to authorized users.

## Introduction

Clinical variation in healthcare describes differences in healthcare practice, processes or outcomes for reasons such as the complexity of a patient’s illness, the burden of illness in different populations, or administrative differences, such as different coding of the same issue [1]. Such variations are evident throughout healthcare systems and services internationally and reflect natural differences between the individuals and population groups receiving care.

Clinical variation in ovarian cancer care has been reported internationally [2–4]. Variation from 5 to 55% has been reported in the receipt of neoadjuvant chemotherapy for women with stage IIIC and IV epithelial ovarian cancer in hospitals that have high volume of both surgery and chemotherapy for ovarian cancer [2]. A population based study from Australia found variation in the provision of standard chemotherapy treatment for women older than 70 years with ovarian cancer, with 68% of these women not receiving standard chemotherapy [5]. Variation in surgical staging, including adherence to guidelines, was found in a Canadian study, with only 44% of gynaecologic surgeons in Ontario adhering to guidelines for surgical staging [3].

Whilst clinical variations are to be expected and are not always problematic, they have attracted increasing interest in health systems internationally as a mechanism for understanding the quality and appropriateness of care provided to patients, highlighting quality features such as efficient, effective and timely care [6]. The dominant conceptual framework and related theory is that set out by Wennberg and colleagues. Wennberg’s classification of variation identifies three categories that can be used to identify when clinical care variation is unwarranted: effective care, preference sensitive care and supply sensitive care [7, 8]. The category of effective care identifies those services and procedures that have been proven effective in the research literature for all patients. Clinical variation related to effective care is often conceptualised through deviation from clinical guidelines.

In ovarian cancer care there are a number of international guidelines that guide effective treatment internationally. The National Comprehensive Cancer Network (NCCN) in the United States initially developed a clinical practice guideline for ovarian cancer in 1996, since then providing regular evidence and consensus based updates, with the most recent guideline published in 2017 [9]. The National Institute for Health and Care Excellence, initially set up in the United Kingdom (UK) in 1999 to reduce variation in the availability and quality of treatment and care in the National Health Service, developed a clinical guideline for clinical practice in ovarian cancer in 2011 [10]. The European Society for Medical Oncology has also published clinical practice guidelines on the management of newly diagnosed and relapsed ovarian cancer [11]. There have been recent developments in peri and intra operative surgical care with the development and implementation of Enhanced Recovery After Surgery Guidelines across a number of surgical areas, including gynaecologic oncology procedures [12, 13].

To date, there has been no synthesis of evidence regarding the degree of unwarranted clinical variation (deviation from effective care) in ovarian cancer care. Data regarding the effectiveness of attempts to reduce unwarranted clinical variation in ovarian cancer care are also lacking. This review aims to address these knowledge gaps by providing a synthesis of evidence in relation to the following questions: (1) What is the evidence of guideline adherence in ovarian cancer and its deviation?; (2) What are the key factors associated with variation in guideline adherence in ovarian cancer care?; (3) What quality improvement approaches been used and what is the evidence of their effectiveness in enhancing guideline adherence in ovarian cancer care?

## Methods

This review was conducted and reported in accordance with the Preferred Reporting Items for Systematic Reviews and Meta-Analysis guidelines [14]. In this study we have defined safe care using Wennberg’s effective care category as rationale for focusing on guideline adherent care and the deviation from these [8].

### Eligibility criteria

Published journal articles reporting studies of any design, including literature reviews, were eligible for the study if they were available in English and published in the last 11 years (2007 to November 2018). This timeframe was chosen to ensure the inclusion of journal articles relevant to contemporary healthcare. To be eligible, studies had to focus on female adults (over the age of 18 years) who had received a diagnosis of primary ovarian, fallopian tube or peritoneal cancer. To be included, the reported outcomes had to focus on guideline adherence and its deviation, the key factors associated with variation in guideline adherence and what quality improvement approaches have been used and evidence of their effectiveness. Conference abstracts, editorials, commentaries and grey literature were excluded. Studies focussed on the centralisation of ovarian cancer care have been defined for the purpose of this review as effective care, using Wennberg’s classification of variation [7, 8]. These studies have been reported on in a previous Cochrane review and have been excluded as out of scope for this study [15].

### Data sources

Keywords and synonyms for the major concepts of ovarian cancer, clinical variation and guideline adherence were developed and combined to form the search strategy. Systematic searches of MEDLINE, EMBASE, SCOPUS and web of science were completed for publications between January 2007 to November 2018. Results were merged using reference management software (endnote; Thomson Reuters), with duplicates removed. The search strategy has been included in Additional file 1. The search terms used in the Medline database search were: Ovarian cancer, ovarian neoplasms, patterns of care, guideline or guideline adherence, variation of care, clinical variation, referral and consultation, referral pathway, optimal care, and framework. The terms framework and guideline were removed for search in web of science and SCOPUS due to the volume of material returned. Reference lists were also scanned to identify additional relevant studies

### Study selection and data extraction

One author (KW) screened the titles and abstracts of the extracted papers, identifying papers that met the inclusion criteria. A random sample of the papers (10%) were screened by a second author (RH) who independently applied the inclusion criteria. The same process was followed for the full text review. Disagreements were resolved through consensus. The following data were extracted from the studies that met the inclusion criteria: author(s), date, method, data source, country, setting, sample/participants, objective, main findings and limitations.

### Data analysis

Data were synthesised using a narrative synthesis approach [16]. This approach was chosen to allow for a text based analysis across the diverse range of research and research approaches identified in this study. The elements of narrative synthesis include developing a theory in light of the findings from the studies, developing a preliminary synthesis, exploring relationships within and between studies, and assessing the robustness of the synthesis [16].

## Results

**Fig. 1:**
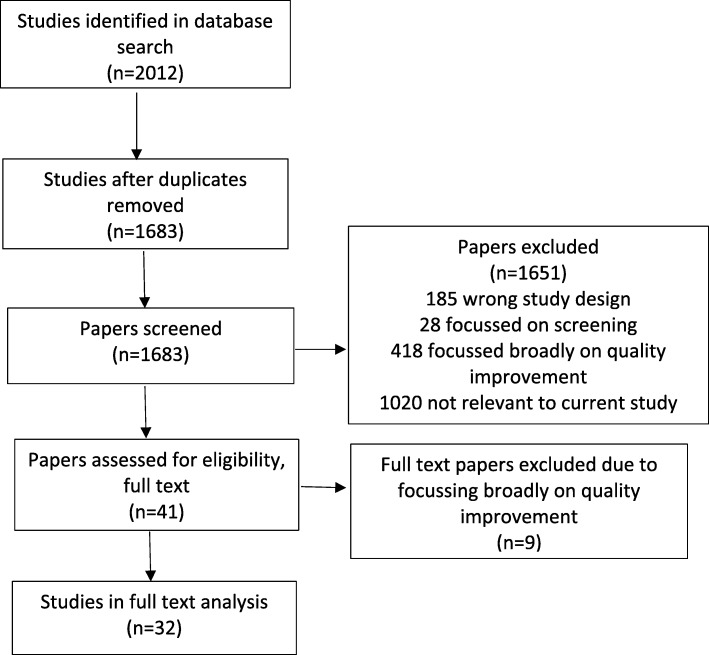
Flow diagram of selection process. Flow diagram outlining the selection process of studies found in the initial search and how studies were excluded, leading to the final 32 studies included in the systematic review

A total of 2012 papers were identified during the search, after removing duplicates 1683 studies were left. Screening of titles and abstracts found 41 studies that required full text review, with 32 studies meeting the inclusion criteria for inclusion in the final review. A flow diagram outlining the results of the search strategy is provided in Fig. 1.

### Excluded studies

Of the 1651 studies excluded, 185 were excluded due to wrong study design, 28 were excluded due to focussing on cancer screening and 427 were excluded as they focused on quality improvement broadly and did not specifically focus on guideline adherence and clinical variation.

### Characteristics of the studies included

Of the 32 studies included in this analysis, 19 reported retrospective population registry or local registry analysis, seven reported retrospective medical record audits, four were literature reviews, one reported a survey with clinicians and the final study included both a literature review and medical record audit. There were no qualitative studies that met the inclusion criteria. The studies were primarily from the United States (17 studies) and Europe (12 studies), with two from Canada, and one from South Korea.

Using a narrative synthesis approach to analyse the 32 papers, three broad groupings of publications were identified: 1) adherence to and deviation from guidelines (11 studies which were local, national or international) [3, 17–26]; 2) factors impacting guideline adherence (12 studies) [27–38] and 3) quality improvement approaches (9 studies) [39–47].

### Guideline adherence and deviation

Of the 11 studies grouped under guideline adherence and deviation, 8 utilised the NCCN ovarian cancer guidelines as the comparator guideline, with 3 using their own local or national guidelines [3, 17–26]. These studies collectively indicated variation in adherence to guidelines for both surgical staging and receipt of chemotherapy. Bristow et al. reported that across all ovarian cancer cases in the Californian Cancer Registry, NCCN adherence was 37.2% over a 7 year period [17]. This compares to Phippen et al. who investigated NCCN guideline adherence in a low volume institute and reported that 85.4% of patients received NCCN guideline adherent surgery over an 8 year period [23]. One of the key factors distinguishing these studies was that in the Phippen et al. study all surgery was completed by gynaecological oncologists, with surgeon type not reported in the Bristow et al. study [17, 23]. Erickson et al. found a higher rate of guideline adherent care, at 78.5%, in their single institution only study [19]. This is in contrast to the population study by Hodeib et al. using data from the Californian Cancer Registry, finding guideline adherence of 24% [21]. See Table 1 for characteristics of the studies included under guideline adherence and deviation.

**Table 1:** Characteristics of included studies (*n* = 32)

Reference	Date	Method	Sample	Objective	Main findings
Aletti et al.	2009	Retrospective medical record audit	All women diagnosed with FIGO stage IIIC primary epithelial ovarian or tubal carcinoma from January 2000 to December 2003 and January 2006 to December 2007	To evaluate the effectiveness of a quality improvement program on surgical quality and the rates of specific procedures.	Data were analysed from a 3-year period prior to the quality improvement (QI) program implementation, and a 3-year period following the implementation. Complete debulking increased from 31% in the pre-QI program to 43% post QI program.
Aletti et al.	2017	Review of NCCN guidelines	NCCN guidelines and previously published quality indicators	To describe existing surgical quality indicators for early and advanced ovarian cancer in relation to the recent NCCN guidelines.	No changes or additions recommend to the indicators. Highlighted the need for the implementation of quality control programs, with the focus on improving surgical outcomes for women with ovarian cancer.
Bristow et al.	2009	Retrospective population based registry analysis	Adult women who underwent a surgical procedure for ovarian cancer in Maryland, USA, from 1 July 2000 to 30 June 2008	To investigate patterns of primary surgical care for ovarian cancer in Maryland according to surgeon and hospital volume.	Surgeon volume was defined as: low ≤4 cases/yr., intermediate 5–9 cases/yr., high ≥10 cases/yr. Hospital volume was defined as: low ≤9 cases/yr., intermediate 10–19 cases/yr., high ≥20 cases/yr. Annual surgeon volume was statistically significant for association with risk of in hospital death. Hospital volume was not found to be statistically significant for association with in hospital death.
Bristow et al.	2009	Retrospective population based registry analysis	Adult women who underwent a surgical procedure for ovarian cancer in Maryland, USA, from 1 July 2000 to 30 June 2009	To evaluate the impact of surgeon and hospital case volume on in-hospital mortality, extent of surgery, length of stay and hospital cost of care.	Surgeon volume was defined as: low < 10 cases/yr., high ≥10 cases/yr. Hospital volume was defined as: low < 20 cases/yr., high ≥20 cases/yr. Surgery performed by a high volume surgeon was associated with a 69% reduction in risk of in-hospital death. Surgery at a high volume hospital was a significant and independent predictor of cytoreductive surgery.
Bristow et al.	2010	Retrospective population based registry analysis	Women over 18 yrs. with FIGO stage IIIc/IV epithelial ovarian cancer from 1996 to 2005 in the USA	To examine and quantify the effect of hospital procedure volume on overall care and processes for women with epithelial ovarian cancer.	Hospital volume was defined as: low < 9 cases/yr., intermediate 9–20 cases/yr., high 21–35 cases/yr., very high > 35 cases/yr. 5-year survival for patients treated at hospitals completing < 21 cases/yr. was significantly poorer than for those treated at hospitals completing > 21 cases/yr.
Bristow et al.	2013	Retrospective population based registry analysis	Cases of epithelial ovarian cancer reported to the California Cancer Registry from January 1999 to December 2006.	To validate NCCN ovarian cancer guideline adherence as a quality process in California, USA.	37.2% of patients received NCCN guideline adherent care. Receipt of non-NCCN guideline based care led to more than a 30% increase in ovarian cancer related death.
Chan et al.	2008	Retrospective population based registry analysis	Women residing in Northern California diagnosed with stage IC/II primary invasive epithelial ovarian cancer from 1 January 1994–31 December 1996.	To investigate factors associated with women under 55 years of age with early stage ovarian cancer not receiving chemotherapy based on standard treatment guidelines.	78.5% of patients with early stage disease received adjuvant chemotherapy. Non-receipt of adjuvant chemotherapy was found to be more likely for those living in poor neighbourhoods, have low grade cancers and be less likely to have seen a gynaecological oncologist.
Cliby et al.	2015	Retrospective population based registry analysis	Women with invasive epithelial ovarian cancer diagnosed between 1 January 1998 and 31 December 2008 in the USA	To evaluate the patterns of ovarian cancer care in the USA to define the influence of patient and institutional factors on overall survival, including the independent relationship between volume and outcomes.	Annual hospital volume was ranked in quartiles: 1–7, 8–16, 17–28 and > 29 cases/yr. 43% of patients received NCCN guideline adherent care. Non-receipt of NCCN guideline care was associated with worse overall survival (HR 1.4, 95% CI 1.36–1.45) with overall survival best in centres with > 29 cases/yr. (HR 0.91, 95% CI 0.86–0.96).
Dodge et al.	2007	Survey	Gynaecologists performing gynaecologic surgery in Ontario	To quantify the gap between current practice in surgical staging of ovarian cancer with Canadian clinical practice guidelines.	44.3% of participants indicated they would complete surgical staging in line with the Canadian clinical practice guidelines. 81% of gynaecological oncologist’s vs 41.5% of non-oncologists indicated they would complete optimal staging.
Erickson et al.	2014	Retrospective medical record audit	Women diagnosed with stage IC/II epithelial ovarian cancer between 2004 and 2009 treated in University of Alabama health system	To examine the reasons preventing patients from receiving NCCN guideline adherent care.	78.5% of patients received NCCN guideline adherent care. All patients were seen by a gynaecological oncologist, removing the bias of physician specialty. Most common reason for not receiving NCCN guideline adherent care was comorbidity.
Galvan-Turner et al.	2015	Retrospective population based registry analysis	Women with stage I-IV epithelial ovarian cancer diagnosed in California between 1 January 1996 and 31 December 2006	To evaluate the feasibility of developing an observed-to-expected (O/E) ratio of adherence to NCCN guidelines as a risk-adjusted hospital measure of quality ovarian cancer care, correlated with disease specific survival.	Care at high O/E hospitals was associated with significant improvement in ovarian cancer specific survival when compared to intermediate O/E and low O/E hospitals. It was found to be feasible to develop a risk adjusted hospital measure of quality.
Harter et al.	2011	Retrospective local quality assurance database analysis	Women with stage IIB-IV ovarian cancer who received surgery between 1997 and 2008 at HSK Hospital, Germany.	To improve the quality of surgery, specifically increase optimal cytoreduction, a quality management program was introduced in 2001. This study reports the outcome of this quality management program in women with advanced ovarian cancer.	Complete resection rates increased from 33 to 62%, with postoperative residuals ≤1 cm increased from 65 to 86%, with residual disease > 1 cm decreasing from 35 to 14%. There was a significant improvement in overall survival from 26 months (1997–2000), to 37 months (2001–2003), to 45 months (2004–2008).
Hodeib et al.	2015	Retrospective population based registry analysis	Women with stage I/II invasive epithelial ovarian cancer diagnosed in California between 1 January 1996 and 31 December 2006	To investigate the impact of socioeconomic status and other demographic variables on adherence to NCCN guidelines in patients with stage I/II disease.	24% of patients received NCCN guideline adherent care. 16% of patients in the lower socioeconomic group received NCCN guideline adherent care.
Ivanova et al.	2017	Retrospective population based registry analysis	Women in Bulgaria with ovarian cancer diagnosed from 2009 to 2011	To investigate if low hospital volume contributes to the number of cases with unspecified morphologic characteristics, which has been assumed as a possible indicator of suboptimal care.	Hospital volume was defined as: low < 30 cases/yr., high ≥30 cases/yr. 53% of patients were treated in low volume hospitals. Low volume vs high volume hospitals had higher number of cases with unspecified grade (27.7% vs 14.3%) and unspecified stage (37.9% vs 27.4%).
Kommoss et al.	2009	Retrospective and prospective local institution clinical tumour registry analysis	Women with stage IA-IIIA ovarian cancer who received surgery between 1997 and 2007 at HSK Hospital, Germany.	To improve the quality of surgery, specifically increase optimal cytoreduction, a quality management program was introduced in 2001. This study reports the outcome of this quality management program in women with early ovarian cancer.	Women receiving standard surgery increased from 27 to 88.5% following the implementation of the quality management program.
Lee et al.	2015	Retrospective medical record audit	Patients with stage I epithelial ovarian cancer treated surgically from January 1991 to December 2010 at Seoul National University Hospital	To evaluate the effects of NCCN guideline adherence on survival outcomes in early stage epithelial ovarian cancer.	Guideline adherent surgical staging was completed in 26.7% of patients, of these 100% received guideline adherent chemotherapy. Difference in disease-specific survival between the two groups was not statistically significant. Recurrence-free survival showed a statistically significant improvement in the guideline-adherent group.
Liang et al.	2015	Retrospective medical record audit	Consecutive patients who underwent primary surgical staging/cytoreduction for ovarian cancer by 6 gynaecologic oncology providers at the Ohio State University from January 2010 and December 2012.	To evaluate compliance at a single National Cancer Institute-designated Comprehensive Cancer Centre with the 8 ovarian cancer quality indicators proposed by the Society of Gynaecology.	60% of patients were completely staged, with lymphadenectomy being the most frequently omitted staging procedure. Bilateral para-aortic lymph node dissection was excluded in 23.6% and bilateral pelvic lymph node dissection excluded in 18.2% of stage I-IIIB cases. 51.8% of optimally debulked stage III patients received intra-peritoneal chemotherapy within 42 days of cytoreduction. Of note, there were documented reasons for decision not to proceed with intra-peritoneal chemotherapy in all but 2 cases. Compliance was reasonable for the other quality indicators.
Mandato et al	2013	Retrospective medical record audit	All patients with a diagnosis of epithelial ovarian cancer in Emilia-Romagna Hospitals, Italy, from 2007 to 2010	To investigate the management and treatment of epithelial ovarian cancer in Emilia-Romagna Hospitals.	Hospital volume was defined as: low ≤10 cases/yr., medium 11–20 cases/yr. and high ≥21 cases/yr. 46% of patients were treated in a high volume hospital. Complete cytoreduction was achieved in 20.1% of patients with stage III-IV disease. Patients treated in high volume hospitals presented a significantly lower risk of dying compared to patients treated in medium and low volume hospitals.
Marth et al.	2009	Retrospective population based registry analysis	Women with ovarian cancer treated in Austria from 1999 to 2004 from participating Austrian gynaecological departments.	To evaluate factors predicting overall survival, with consideration of department volume.	Hospital department volume was defined as: small ≤23 patients/yr., large ≥24 patients/yr. Survival was longer at large vs small departments, 5-year survival of 69% vs 61% (*p* = 0.01). Department size was found to be an independent predictor of survival (HR 1.39 for treatment in small departments).
Phillips et al.	2017	Retrospective medical record audit and literature review	Patients diagnosed with stage III/IV advanced ovarian cancer from 16 August 2007 to 3 February 2014 at the Pan-Birmingham Gynaecological Cancer Centre, UK.	To evaluate the effect of the denominator on survival of the total advanced ovarian cancer cases identified through a systematic literature, as well as data from the Pan-Birmingham Gynaecological Cancer Centre, UK.	Reporting the denominator in studies in advanced ovarian cancer is important to correctly interpret the outcomes being reported. Of the 18 studies identified, 2 reported on their total patient cohort, with no studies reporting overall survival for their total patient cohort. Data from the medical record audit demonstrated decreasing overall survival as treatment became less aggressive. Median overall survival for the total patient cohort was 30.2 months.
Phippen et al.	2013	Retrospective medical record audit	Patients diagnosed with epithelial ovarian cancer, fallopian tube cancer, or primary peritoneal cancer between 2002 and 2010 at Brooke Army Medical Centre.	To evaluate the optimal cytoreduction rate, NCCN guideline adherence rate and surgical outcomes at a low volume institution.	85.4% of patients received NCCN guideline adherent surgery. Compliance rate in line with reports from high volume centres from this low volume centre.
Querleu et al.	2013	Literature review and expert consensus	Development of quality indicators based on standards of practice and expert consensus.	Development of quality indicators for France by the French Society of Gynaecologic Oncology.	Three structural, eight process and two outcome quality indicators were developed. The paper highlighted the need to implement the indicators through a quality assurance program and pose that their implementation would result in a positive impact in survival for women with ovarian cancer in France.
Querleu et al.	2016	Literature review and expert consensus	5 initiatives publishing quality indicators for advanced ovarian cancer surgery were identified. A multidisciplinary International Development Group was established to develop the quality indicators.	To develop quality indicators for advanced ovarian cancer surgery, carried out by the European Society of Gynaecologic Oncology (ESGO).	10 quality indicators were developed through this evidence based and consensus process. The indicators are categorised as structural, process or outcome. ESGO aims to implement their quality assurance program for advanced ovarian cancer using these indicators, with achievement targets applied to each indicator.
Shakeel et al.	2017	Retrospective population based registry analysis	Women with an ovarian cancer diagnosis in Canada (excluding Quebec) from 2004 to 2012.	To evaluate the quality of surgical care for women with ovarian cancer in Canada by assessing surgical volume and surgeon speciality on short-term postoperative outcomes.	Hospital volume was defined as: low 1–27 procedures/yr., intermediate 29–28 procedures/yr., high 99–201 procedures/yr. Surgeon volume was defined as: low 1–4 procedures/yr., intermediate 5–23, high 24–57 procedures/yr. In-hospital mortality rate in 2004 was 1.3%, declining to 0.74% in 2012. Increasing age and comorbidity were significant predictors of in-hospital mortality. Hospital surgical volume was a significant predictor of reduced risk of in-hospital mortality and failure-to-rescue, but longer length of stay. Surgeon volume significantly predicted an increased risk of major complications and prolonged length of stay. Ovarian cancer surgery in Canada is performed by a large number of surgeons with low surgical volume/yr.
Sijmons et al.	2007	Retrospective medical record audit	Women diagnosed between 1991 and 1997 with stage IA, IB or IC ovarian cancer in the central region of the Netherlands.	To assess compliance with current surgical staging and adjuvant local treatment guidelines and overall survival for patients with early stage epithelial ovarian cancer.	32.8% of patients received optimal surgical staging, chemotherapy guidelines were followed in 100% of grade 1, and 74.6% of grade II and III patients. 5-year overall survival in the optimally staged cohort was 97.6, and 68.5% in the non-optimally staged cohort.
Sobrero et al.	2016	Retrospective medical record audit	Residents of the Piedmont Region of Italy diagnosed with ovarian cancer in 2009	To investigate whether ovarian cancer in the Piedmont Region is being managed according to current local, evidence-based clinical guideline; to identify determinants of lack of adherence to guidelines; and to evaluate the association between adherence to clinical guidelines and survival.	35.2% of patients received guideline adherent surgery, with 87.8% of patients receiving guideline adherent chemotherapy. Receipt of guideline adherent chemotherapy was associated with a significant reduction in all-cause mortality (HR, 0.5; 95% CI,0.28–0.89)
Uppal et al.	2018	Retrospective population based registry analysis	Women with a diagnosis of high-grade serous ovarian carcinoma undergoing primary cytoreductive surgery from 2004 to 2013.	To evaluate the role of 30-day readmission rate as an indicator of quality of care in ovarian cancer surgery.	Hospital case volume was defined as: ≤ 10 cases/yr., 11–20 cases/yr., 21–30 cases/yr., > 31 cases/yr. Higher volume hospitals had higher 30-day readmission rates, but had significantly lower 30 (OR 0.69, 95%CI 0.50–0.96) and 90-day mortality (OR 0.74, 95%CI 0.60–0.91). Higher volume hospitals had higher re-admission rates, but also had higher NCCN guideline adherence, and improve 5-year overall survival.
Verleye et al.	2009	Literature review and expert consensus	The PUBMED database was searched, with journal papers as well as guidelines and standards of care reviewed. Details of the search results were not reported.	To develop a list of process quality indicators for staging laparotomy in stage I-IIIA ovarian cancer, and debulking laparotomy in stage IIIB-IV ovarian cancer for the European Organisation for Research and Treatment of Cancer (EORTC-GCG).	5 indicators were proposed for staging laparotomy (stage I-IIIA), with 6 indicators proposed for debulking surgery (stage IIIB-IV). These proposed indicators will need to be evaluated for feasibility, validity and reliability.
Warren et al.	2017	Retrospective population based registry analysis	Women in the USA diagnosed with ovarian cancer in 2002 and 2011	To evaluate population based trends in ovarian cancer treatment and survival, with a focus on NCCN guideline adherent care, assessing patient and provider characteristics and estimating trends in 2-year cause-specific survival.	In stage II ovarian cancer the percent of women who received guideline adherent surgery increased from 18.3% in 2002 to 31.7% in 2011, in stage II from 48.9% in 2002 to 56.7% in 2011, with stage IV remaining stable at 34% for the two time periods. Receipt of guideline adherent chemotherapy rose significantly over the time period, with at least 70% of women receiving multiagent chemotherapy in 2011.
Wright et al.	2013	Retrospective population based registry analysis	Women in the USA aged 65 years or older with ovarian or uterine cancer who underwent surgery from 2000 to 2007.	To estimate trends in hospital volume and referral patterns for women with uterine and ovarian cancer.	The median hospital volume remained constant throughout the study period at 2 cases per 2 years. During the study period there was market concentration, where a similar number of patients undergo the procedure over time, with the number of hospitals decreasing, with increasing number of procedures being completed in higher volume hospitals. Overall, women with gynaecologic cancers continue to be treated at very low volume centres.
Wright et al.	2012	Retrospective population based registry analysis	Women in the USA aged 18–90 with ovarian cancer and underwent oophorectomy from 1998 to 2009.	To examine the influence of failure to rescue as a source of variation in mortality for patients with ovarian cancer.	Hospital volume was defined as: low 1–36 procedures/yr., intermediate 36.1–53 procedures/yr., high > 53 procedures per year. The overall complication rate was 22.8%, with the failure to rescue rate being 6.2%, decreasing from 8.7% in 1998 to 6% in 2009. The adjusted failure to rescue rate was 48% higher at low compared to high volume hospitals.
Wright et al	2017	Retrospective population based registry analysis	Women in the USA diagnosed with invasive epithelial ovarian cancer from 2004 to 2013	To examine if is lower volume hospitals that comply with NCCN quality metrics can achieve outcomes similar to high volume hospitals.	Five quality metrics were defined based on NCCN guidelines, with adherence to the metrics identified, reported on and aligned to hospital volume. Compliance with the quality metrics increased with hospital volume. For each volume category 2-year adjusted survival increased with adherence to the quality metrics, from 61.4% in the low volume with low quality metric compliance hospitals to 78.6% at the highest volume and high quality metric compliance hospitals.

### Factors impacting guideline adherence

Individual hospital and/or surgeon volume and their link to improved outcomes has been identified for surgery that is technically complex [48]. Hospital and/or surgeon volume emerged as the primary factor associated with the degree of guideline adherence identified in 11 of the included studies in this review that in turn was linked to ovarian cancer outcomes. Eight of the 11 studies identified higher volumes with improved outcomes at a national population level, with three studies focussing on higher volume leading to improved outcomes at a state or specific geographical level [27–32, 34–36, 38]. Survival at both 5-years and 4-years was found to be better for women treated in higher volume hospitals [29, 30, 32, 38]. The Marth et al. and Bristow et al. studies found hospital volume was an independent predictor of survival [29, 36]. Hospital volume was also found to be a statistically significant and independent predictor of optimal cytoreductive surgery, i.e. guideline adherent care [28, 29]. Shakeel et al. and Wright et al. reported that higher volume hospitals had lower in-hospital mortality and morbidity [33, 34]. The study by Uppal et al. investigated the use of 30-day readmission following cytoreductive surgery as a measure of quality [49]. This study found that although higher volume hospitals had higher rates of 30-day readmission, they also had higher rates of guideline adherent care and an improved 5-year survival when compared to lower volume hospitals with lower 30-day readmission rates [37]. This supports their proposal that 30-day readmission following ovarian cancer surgery is not a robust measure of quality care. Overall the studies found that improvement in guideline adherence was associated with improved outcomes in hospitals where there was high volume ovarian cancer care at a hospital and/or surgeon level. See Table 1 for characteristics of the studies included under factors impacting guideline adherence.

### Quality improvement approaches

There were nine studies identified investigating quality improvement approaches that attempted to detect, respond to or address clinical variations. Five of the studies focussed on the development of quality indicators for guideline adherent care as a way to identify variation in clinical practice and improve outcomes [40, 44–47]. Three studies reported on the impact of quality improvement programs on improving outcomes in ovarian cancer through increasing adherence to clinical guidelines, resulting in a reduction of unwarranted clinical variation [39, 41, 42]. The final study reported on one hospital’s compliance with Society of Gynecologic Oncology quality indicators [43]. Five studies reporting on the development of quality indicators all had similarities in the indicators developed, with a focus on rates of complete surgical resection [40, 44–47]. Three of these studies focussed further on the type of surgeon and their surgical volume [40, 45, 47]. The study by Phillips et al. focussed on the importance of identifying the denominator in advanced ovarian cancer studies and quality indicators, to ensure outcomes are able to be appropriately interpreted and the ‘denominator effect’ is understood [44]. Phillips et al. defined the denominator in their study as the total number of advanced ovarian cancer cases presenting or referred to the cancer centre [44]. This then allowed them to understand the proportion of patients who received treatment and the cancer centres overall survival rates for women with advanced ovarian cancer [44]. The study by Aletti et al. found an increase in complete cytoreductive surgery from 31 to 43% following the implementation of a quality improvement program [39]. The quality improvement program implemented included comparison of hospital results with the latest evidence, confidential surgeon benchmarking, educational sessions for trainees on new and complex surgical techniques and improving the availability of experienced staff members to assist with complex surgical cases [39]. The study by Kommoss et al. found women with stage 1A-IIIA ovarian cancer had an improvement of 27 to 88.5% in complete cytoreductive surgery following the implementation of a quality improvement program [42]. Harter et al. further reported on the same quality improvement program for women with later stage disease, finding complete cytoreductive surgery rates for women with stage IIB-IV ovarian cancer improved from 33 to 62%, with overall survival improving from 26 months to 45 months following the implementation of the quality improvement program [41]. The quality improvement program reported on in the Kommoss et al. and Harter et al. studies focussed on optimal surgical staging, receipt of platinum based chemotherapy, surgeon volume and experience, as well as the implementation of dedicated ovarian cancer surgical teams, benchmarking of morbidity and outcomes, and improvement of interdisciplinary assessment and management [41, 42]. There are commonalities between the three studies that reported on outcomes of the implementation of quality improvement programs, including adherence to guideline based care, improvement in ongoing surgical education and surgeon benchmarking [40–42]. The study by Liang et al. found high clinical variation in the indicators of complete surgical staging and the administration of intraperitoneal chemotherapy within 42 days of optimal cytoreductive surgery [43]. See Table 1 for characteristics of the studies included under quality improvement approaches.

## Discussion

This review completed a synthesis of the evidence in relation to the degree of unwarranted clinical variation for women with ovarian cancer and the effectiveness of attempts to improve clinical variation. Findings from the 32 included studies were extracted in relation to three key areas: adherence to and deviation from guidelines, factors impacting guideline adherence, and quality improvement approaches to reduce variation through enhanced guideline adherence. Despite strong evidence of the optimal care process for ovarian cancer, aligning with Wennberg’s classification of effective care, variation was apparent in the extent to which guideline adherent care was practiced for women with ovarian cancer internationally [8]. Commonalities between the studies included non-adherence to guidelines in surgical staging, optimal cytoreductive surgery and receipt of adjuvant chemotherapy [3, 16–24]. Variation in guideline adherent care, and specifically deviation from effective care, is likely to lead to unwarranted clinical variation in outcomes for ovarian cancer patients. Quality improvement approaches such as the use of quality indicators and team-based quality improvement projects have been utilised to reduce unwarranted clinical variation, with some evidence of success. The most successful quality improvement approaches found in this review focused on the development of quality improvement programs across gynaecological oncology services. These successes facilitated clinical change through the implementation of dedicated ovarian cancer surgical teams, education on new and emerging surgical techniques and benchmarking at a hospital and surgeon level [38, 40, 41].

Surgical volume at both a hospital and surgeon level have been associated with improved outcomes in ovarian cancer for many years, with the study by Luft et al. in 1979 being one of the earliest studies to identify this association [48]. The association between improved surgical outcomes and high hospital or surgeon volume has been identified in a number of cancer types that require technically complex surgery, such as pancreatectomy [48, 50]. One of the key factors in the relationship between volume and care outcomes is thought to be the increased likelihood of those practicing high volume care to adhere to best practice guidelines. Our findings identified evidence to support the relationship between hospital volume, surgeon volume, guideline adherence and outcomes in ovarian cancer care such that higher volume hospitals and surgeons were found to be independently associated with increased survival, as well as increased levels of guideline adherent care [27–36]. Guideline adherent surgery in ovarian cancer includes complex surgical techniques that a specialist surgeon, such as a gynaecological oncologist, may only achieve competency in though completing a high volume of these surgeries, with the support of a specialist multidisciplinary team in their hospital [15, 29].

A key finding of this review is that the implementation of quality indicators through a formal quality improvement program has led to improvements in guideline adherent care, as highlighted in the improvement of complete cytoreductive surgery in the studies by Aletti et al., Kommoss et al. and Harter et al. [39, 41, 42]. While the development of quality indicators is a key part in this process, the included studies highlighted improvements in clinical variation that can be achieved when indicators are implemented into clinical practice. Three of the studies outlined the process of developing quality indicators at national and international levels [45–47]. However, the four studies that reported on the implementation of quality indicators through quality improvement programs were implemented in single institutions [39, 41–43]. These studies demonstrated that improvements in guideline adherent care could be made, with only one study reporting on an improvement in overall survival [41].

### Implications

Given the relationship between hospital and surgical volume, guideline adherence and care outcomes, our findings suggest that there is value in programs to promote guideline adherent care in hospitals with high surgical volume, at both a hospital and surgeon level [30, 33]. Development and implementation of quality indicators and quality improvement programs to improve ovarian cancer outcomes have been used internationally in an attempt to reduce unwarranted clinical variation in ovarian cancer care [39–43, 45–47]. A core strategy associated with such programs is the centralisation of care to hospitals that have specialist gynaecological oncology centres and complete a high volume of ovarian cancer surgery at both the hospital and surgeon annually [51–54]. Evidence suggests that centralising ovarian cancer care into specialist hospitals improves access to gynaecological oncologists, multidisciplinary cancer care teams and higher surgical volume hospitals and surgeons may lead to improved survival [15].

The development of gynaecological oncology as a surgical sub-speciality in Australia, the UK and North America has led to increasing specialist care in the treatment of gynaecological cancer. In a study identifying practice guideline adherence in Ontario, Canada, 81% of gynaecological oncologists reported completing surgical staging adherent to guidelines, with only 41.5% of non-oncologists completing guideline adherent surgical staging [3]. A study by Chan et al. found treatment by a gynaecological oncologist led to more women in California to undergo primary staging surgery (91.9% vs. 69.1%) and chemotherapy (90% vs. 70.1%) than those treated by a non-oncologist [55].

An important focus for future research is to establish evidence of the effectiveness of the implementation of quality improvement programs, reporting on survival over time. Evidence of the relationship between quality improvement programs and survival is important for assessing the impact of implementing quality indicators through quality improvement programs as a way to decrease unwarranted clinical variation and improve outcomes for women living with ovarian cancer. Analysis of the implementation of quality indicator and quality improvement programs across regional or national jurisdictions that aim to improve outcomes in ovarian cancer beyond a single institution level would be valuable.

### Limitations

The included studies and review process were subject to some limitations. Whilst hospital and surgeon volume were identified as important, a key challenge for interpreting the results of these studies was the range of case volume for hospitals, with each study defining volume in different ways. High hospital volume ranged from > 20 to > 30 cases/yr. [29–32, 36]. High surgeon volume was more consistent, being identified as ≥10 cases/yr. in the two studies by Bristow et al. that investigated surgeon level volume [27, 28]. For health systems undertaking centralisation of ovarian cancer surgery this may lead to challenges in identifying which hospitals should be completing ovarian cancer surgery. Reliance on administrative datasets created limitations such as selection bias, as most registries did not cover the entire population, with a lack of information on stage of disease, extent of cytoreductive surgery and surgeon specialist type. Two examples are the study by Erickson et al. as a single institute study finding a high rate of guideline adherent care, at 78.5%, and the study by Hodeib et al. which used population data and found a low rate of guideline adherent care at 24% [19, 21]. A limitation in study design, using single institution versus population data, may in some way explain the vast difference in guideline adherence between the two studies [19, 21]. The review process may have omitted relevant material by including only studies published in English, the exclusion of studies allocated to broader quality of care issues, as there are many factors associated with clinical variation, guideline adherence being just one, and the date range of the past 11 years.

## Conclusion

This review has reported for the first time exploring guideline adherence and the factors impacting on this towards clinical variation in ovarian cancer, highlighting guideline adherence and deviation, factors impacting guideline adherence, and quality improvement approaches. There is evidence of deviation from effective care in ovarian cancer, demonstrated through deviation from best practice guidelines. Such deviation is likely to lead to unwarranted clinical variation in ovarian cancer care. Centralising care to higher volume centres and surgeons, and the growth of gynaecologic oncology as a specialty appear to be associated with enhanced guideline adherence, reduced variation and better outcomes as a result. The development, implementation and reporting of quality performance programs may also lead to reduced unwarranted variation in ovarian cancer care, but evidence is currently limited regarding the effectiveness of these programs at regional and national levels and on longer term outcomes.

## Additional file

Additional file 1: Search terms. Overview of the search terms used for the four database searches. (DOCX 16 kb)

## Abbreviations

NCCN: National Comprehensive Cancer Network
UK: United Kingdom
