# Supplementary material for: Enhancing ovarian cancer care: a systematic review of guideline adherence and clinical variation
Source: BMC Public Health. 2019 Mar 12;19:296. doi: 10.1186/s12889-019-6633-4 (PMC6416902; doi:10.1186/s12889-019-6633-4)
Supplement: Supplementary file 1 — Search terms. Overview of the search terms used for the four database searches. (DOCX 16 kb) [file 12889_2019_6633_MOESM1_ESM.docx]

**Appendix A: Search terms**

**Medline search terms**

| 1. Ovarian Neoplasms/ |
| --- |
| 2. ovarian cancer.mp. |
| 3. 1 or 2 |
| 4. patterns of care.mp. |
| 5. Guideline/ |
| 6. Guideline Adherence/ |
| 7. guideline.mp. [mp=title, abstract, original title, name of substance word, subject heading word, keyword heading word, protocol supplementary concept word, rare disease supplementary concept word, unique identifier, synonyms] |
| 8. guideline adherence.mp. [mp=title, abstract, original title, name of substance word, subject heading word, keyword heading word, protocol supplementary concept word, rare disease supplementary concept word, unique identifier, synonyms] |
| 9. variation of care.mp. |
| 10. clinical variation.mp. |
| 11. "Referral and Consultation"/ |
| 12. referral pathway.mp. [mp=title, abstract, original title, name of substance word, subject heading word, keyword heading word, protocol supplementary concept word, rare disease supplementary concept word, unique identifier, synonyms] |
| 13. optimal care.mp. |
| 14. framework.mp. |
| 15. 4 or 5 or 6 or 7 or 8 or 9 or 10 or 11 or 12 or 13 or 14 |
| 16. 3 and 15 |
| 17. limit 16 to (english language and humans and yr="2007 -Current") |

**Embase search terms**

| 1. ovary tumor/ |
| --- |
| 2. ovarian cancer.mp. [mp=title, abstract, heading word, drug trade name, original title, device manufacturer, drug manufacturer, device trade name, keyword, floating subheading word] |
| 3. 1 or 2 |
| 4. patterns of care.mp. |
| 5. practice guideline/ |
| 6. protocol compliance/ |
| 7. variation of care.mp. |
| 8. clinical variation.mp. |
| 9. (referral and consultation).mp. [mp=title, abstract, heading word, drug trade name, original title, device manufacturer, drug manufacturer, device trade name, keyword, floating subheading word] |
| 10. referral pathway.mp. |
| 11. optimal care.mp. |
| 12. framework.mp. |
| 13. 4 or 5 or 6 or 7 or 8 or 9 or 10 or 11 or 12 |
| 14. 3 and 13 |
| 15. limit 14 to (english language and yr="2007 -Current") |

**Scopus and Web of Science search terms**

1. Ovarian neoplasms OR ovarian cancer
2. Patterns of care
3. Guideline adherence
4. Variation of care
5. Clinical variation
6. Referral pathway
7. Optimal care
8. 2 OR 3 OR 4 OR 5 OR 6 OR 7
9. 8 AND 1
